# Two-way AIC: detection of differentially expressed genes from large scale microarray meta-dataset

**DOI:** 10.1186/1471-2164-14-S2-S9

**Published:** 2013-02-15

**Authors:** Koki Tsuyuzaki, Daisuke Tominaga, Yeondae Kwon, Satoru Miyazaki

**Affiliations:** 1 Department of Medical and Life Science, Faculty of Pharmaceutical Science, Tokyo University of Science, 2641 Yamazaki, Noda, 278-8510, Japan; 2 Computational Biology Research Center (CBRC), National Institute of Advanced Industrial Science and Technology (AIST), 2-4-7 Aomi, Koto-ku, Tokyo, 135-0064, Japan

## Abstract

**Background:**

Detection of significant differentially expressed genes (DEGs) from DNA microarray datasets is a common routine task conducted in biomedical research. For the detection of DEGs, numerous methods are proposed. By such conventional methods, generally, DEGs are detected from one dataset consisting of group of control and treatment. However, some DEGs are easily to be detected in any experimental condition. For the detection of much experiment condition specific DEGs, each measurement value of gene expression levels should be compared in two dimensional ways, or both with other genes and other datasets simultaneously. For this purpose, we retrieve the gene expression data from public database as possible and construct "meta-dataset" which summarize expression change of all genes in various experimental condition. Herein, we propose "two-way AIC" (Akaike Information Criteria), method for simultaneous detection of significance genes and experiments on meta-dataset.

**Results:**

As a case study of the *Pseudomonas aeruginosa*, we evaluate whether two-way AIC method can detect test data which is the experiment condition specific DEGs. Operon genes are used as test data. Compared with other commonly used statistical methods (*t*-rank/F-test, RankProducts and SAM), two-way AIC shows the highest specificity of detection of operon genes.

**Conclusions:**

The two-way AIC performs high specificity for operon gene detection on the microarray meta-dataset. This method can also be applied to estimation of mutual gene interactions.

## Background

**Figure 1:**
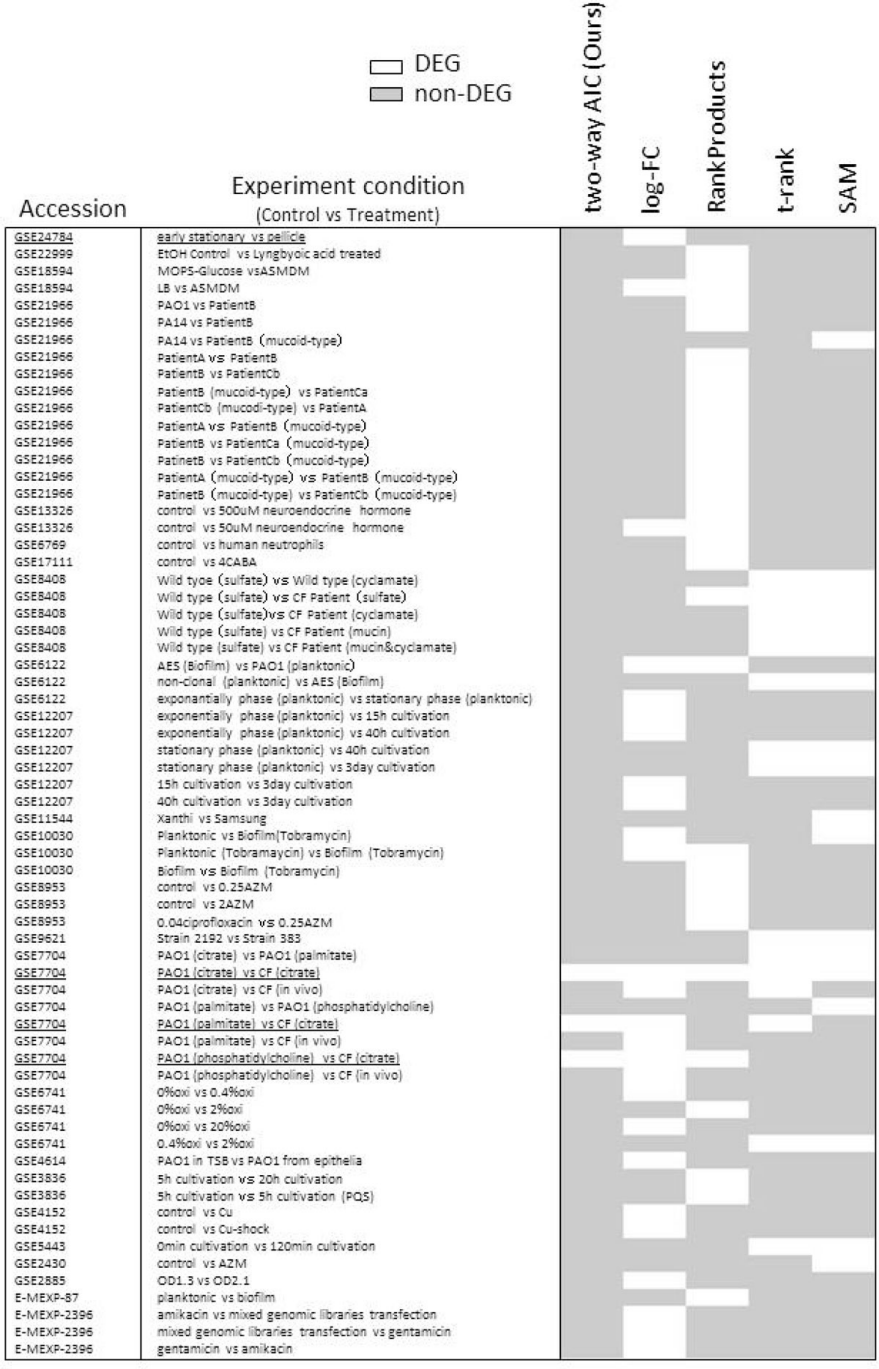
**Expression change of pyoverdin genes**. We analyze some expression data of pyoverdin genes (*pvdD *and *pvdJ*) of public database (GEO and Array- Express) by commonly used statistical methods (log-FC, RankProducts, *t*-rank and SAM). The threshold value of log-FC is set to 2 (4-fold) and that of RankProducts, *t*-rank and SAM are set to upper 300 gene. All dataset are normalized by RMA method separately. If both genes are co-expressed, corresponding box is filled in white, otherwise gray. Figure shows that pyoverdin genes are prone to be detected in any experiment condition and our method focuses on much experiment condition specific DEGs (GSE7704).

Detection of significant differentially expressed genes (DEGs) from DNA microarray datasets is a common routine task conducted in biomedical research [1-3]. For the detection of DEGs, numerous methods are proposed [4-7]. By such conventional methods, generally, DEGs are detected from one dataset consisting of group of control and treatment. However, some DEGs are easily to be detected in very wide or common experimental conditions. For example, "pyoverdin" genes (*pvdD *and *pvdJ*) [8] of *Pseudomonas aeruginosa*, which are ones of Iron transporter proteins and involved in cell division, are generally detected as DEGs in experimental conditions which are conducted to observe cell division (such as GSE24784 in GEO database) (Figure 1). Additionally, in analyses of some expression dataset of public database by commonly used statistical methods, pyoverdin genes are also detected as DEGs in many other experimental condition which are not conducted to observe cell division. Literatures suggested that this may be because of pyoverdin is involved in many other biological processes such as cell-to-cell signaling (Quorum Sensing, QS) [9] and virulence factor production [10]. In this way pyoverdin genes are prone to be detected as DEGs in any experiment condition, however, many researchers may want to these genes to be detected in the special experiments (i.e., cell division condition). For this purpose, each measurement value of gene expression levels should be compared in two dimensional ways, or both with other genes and other datasets simultaneously.

**Figure 2:**
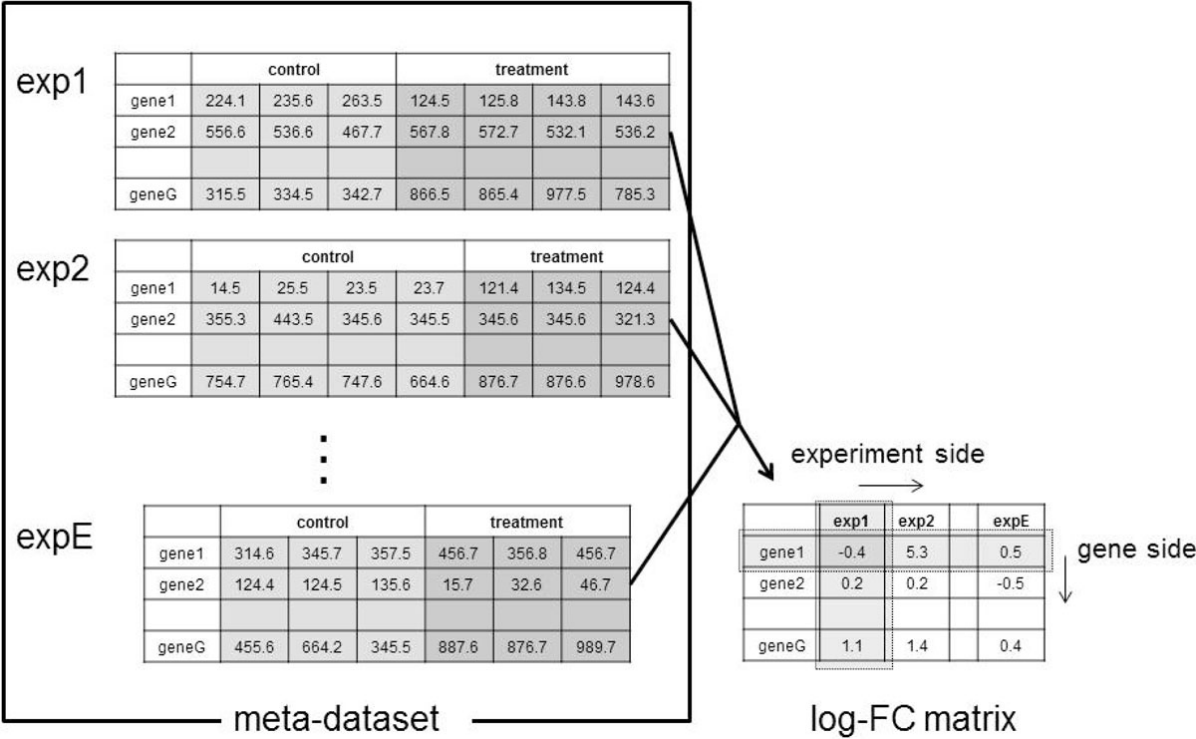
**Meta-dataset and log-FC matrix**. A meta-dataset is a set of multiple datasets. Each dataset consists of a control group and a treatment group, each of which has one or more DNA microarray data. The measured probe (gene) is common to all datasets. The element of *F_i,j _*in log-FC matrix is the log-transformed (base 2) fraction of arithmetic mean values of treatment and control group in *i*-th gene of *j*-th dataset.

For the detection of such DEGs, we retrieve the gene expression data from public database as possible and construct "meta-dataset" which summarize expression change of all genes in various experiment condition (Figure 2). Although there are no 'de fact' standard definition for meta-datasets, log ratio value which are widely used to analyze DNA microarray data can be introduced to construct meta-datasets when each dataset is consist of control and treatment experiment data.

In such meta-datasets, direct application of widely used conventional statistical methods is not suitable to detect two-dimensional DEGs because such methods are intended to find special genes among all experiments to be analyzed.

For example, ANOVA [11-14] is applied very widely for multi-group analysis method, but its concludes only that differences between groups (genes) are significant or not. Therefore ANOVA can not detect simultaneously specific genes in specific experiments as two-dimensional DEGs.

Outlier detection methods are also widely used to detect DEGs, such as Shannon entropy [15] or Sprent's non-parametric method [16]. In difference to ANOVA, these methods can also detect both special genes or special experimental conditions, but it is not simultaneously. It is one-dimensional and similar to ANOVA.

Multiple testing [17] (multiple comparisons, such as Bonferroni correction, Tukey-Kramer's method, and Games-Howell's method) also produce limited results as same as outlier detections. For an example of a dataset consisting of *N *genes and *E *experiments, it never means that the *i*-th gene of the *j*-th experiment is a DEG when multiple testing shows that the *i*-th gene (size *E *vector) is significantly different from other genes and the *j*-th experiment (size *N *vector) is significantly different from other experiments independently. This is because most multiple testing methods are conducted to ascertain differences between mean values of groups.

Herein, we propose "two-way AIC" (Akaike Information Criteria) method for simultaneous detection of significant genes and experiments on metadatasets. This method detects specific genes that are differentially expressed in specific experimental conditions. Here, we present comparison of the performance of our method to other widely used statistical methods and show that two-way AIC method has high specificity for detection of test data which tend to express in specific experiment condition.

## Methods

### Meta-dataset and log-FC matrix

A meta-dataset is a set of plural datasets. Each dataset consists of measurement groups of two kinds: control and treatment. Both control and treatment groups consist of one or more DNA microarray measurements. Genes (probes) are common to all microarrays (Figure 2).

After normalization is applied, we summarized the expression data of each dataset as logarithm of fold change values (log-FC). This step is for removal of systematic bias between samples of different studies [18]. Log-FC of each gene are calculated based on ratios of measurement values of treatment to those of control for each dataset. Log-FC is defined as a logarithm (base 2) of a fraction of arithmetic mean values of treatment and control shown as follows:

(1) $$F_{i,j}=\text{log}\left(\frac{{\bar{t}}_{i,j}}{{\bar{c}}_{i,j}}\right),$$

where ${\bar{t}}_{i,j}$ and ${\bar{c}}_{i,j}$ respectively denote the arithmetic mean values of treatment and control measurements of *i*-th gene of *j*-th dataset (Figure 2). We define the row side direction of the matrix of log-FC values (log-FC matrix) as the "gene side" and the column side direction as the "experiment side".

### Judgment matrix

**Figure 3:**
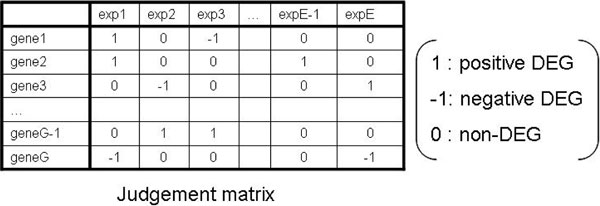
**Judgment matrix**. The judgment matrix is the summary of results of each DEG detection method. This matrix is derived from the meta-dataset or log-FC matrix, where each element has one value: 1 (positive DEG), -1 (negative DEG) or 0 (non-DEG).

Here we define the judgment matrix, which is the conclusion based on results of DEG detections described as a two-dimensional table (gene and experiment) (Figure 3). The element *x_i,j _*in the judgment matrix is the result of DEG detection of the *i*-th gene in the *j*-th experiment (dataset). Each element takes one value out of three values: 1, -1, or 0.1 means positive DEG (specifically higher expression), -1 means negative DEG (specifically lower expression) and 0 means that it is not a DEG. Generally, DEG detection can be performed both gene side and experiment side direction.

### Two-way AIC

Our two-way AIC, based on the *U*-value method [19,20], is applied to the log-FC matrix. It detects DEGs as outliers of both the gene side and the experiment side simultaneously. Given a group of samples, and the *n *furthest samples from the group's average are presumed as outliers, the *U*-value is defined as

(2) $$U=n\text{log}\sigma +\sqrt{2}\times s\times \frac{\text{log}n!}{n},$$

where *n *is the number of outliers, and *σ *and *s *respectively denote the standard deviation and the number of non-outlier samples. Outliers are estimated as the best presumption of outliers which minimizes *U*. In this paper, the search range is restricted to within 25 percent of the number of data.

When the *U*-value method is applied in the gene side direction, specific experiments are detected as outliers for each gene. Similarly, when the *U*-value method is applied in the experiment side, specific genes are detected for each experiment. The detected outliers are described as 1 (positive outlier) or -1 (negative outlier).

**Figure 4:**
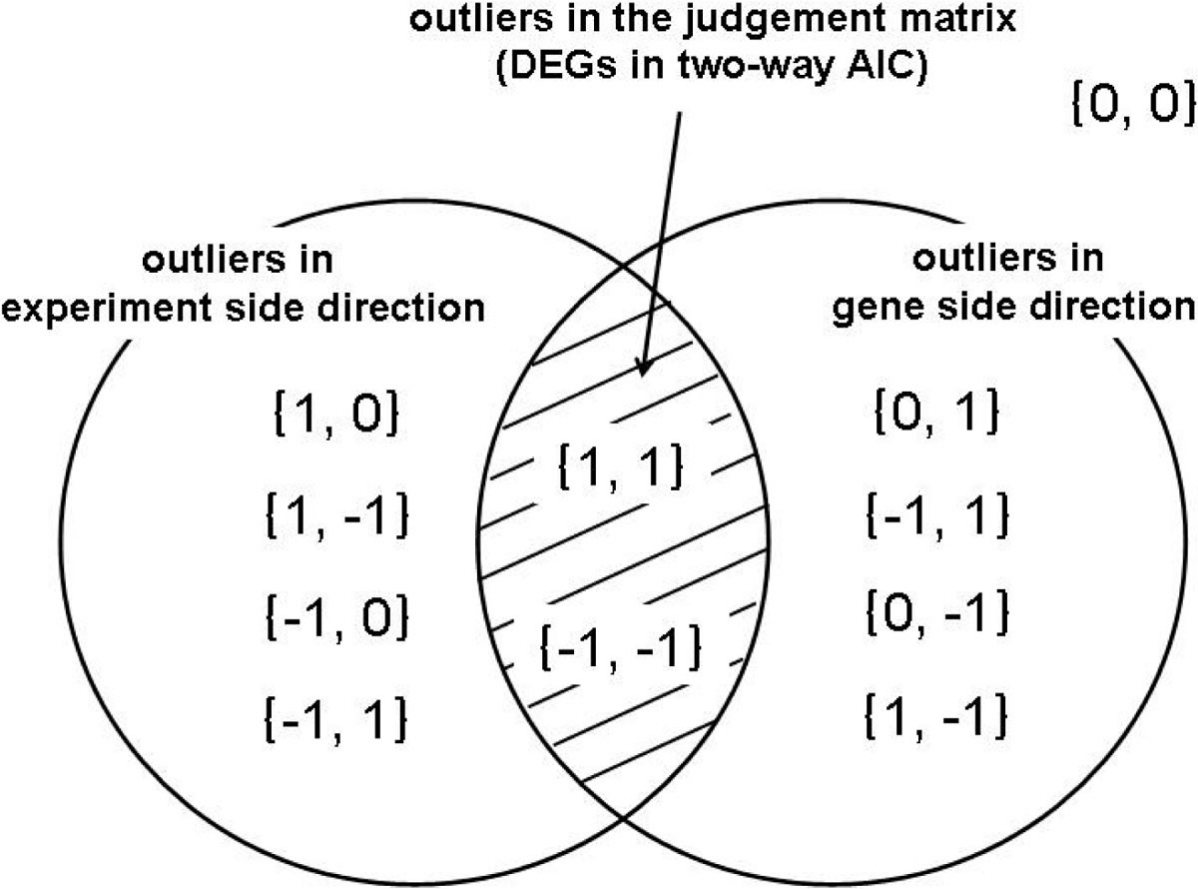
**DEGs in two-way AIC**. Two-way AIC detects DEGs as outliers of the gene side and the experiment side. In each direction, outliers are detected by *U*-value method. Outliers are described as 1 (positive outlier) or -1 (negative outlier). Based on two sets of results for each direction, *x_i,j _*in the judgment matrix is set to the value of the label if two labels are the same. Finally it is judged as a DEG (Figure 3).

Detection results of *i*-th gene of *j*-th experiment have two labels, the result on the gene side and that of experiment side direction. *x_i,j _*in the judgment matrix is set to the value of the label if two labels are the same. Finally it is judged as a DEG (Figure 4). The element (*x_i,j_*) of the judgment matrix of two-way AIC is described as

(3) $$x_{i,j}=\left\{\begin{array}{cc} U_{i,j}^{ex}\cap U_{i,j}^{gn} & \left(U_{i,j}^{ex}=U_{i,j}^{gn}\right)\\ 0 & \left(otherwise\right), \end{array}\right)$$

where $U_{i,j}^{ex}$ is the element on the *i*-th gene, *j*-th experiment in the judgment matrix by Ueda's statistic on the experiment side and $U_{i,j}^{gn}$ is the element on the *i*-th gene, *j*-th experiment in the judgment matrix by Ueda's statistic on the gene side.

## Results

The two-way AIC method is applied to a prokaryote gene expression meta-dataset to demonstrate its detection performance, and it is compared in specificity of detection of test data (operon genes) [21,22], which generally tend to express simultaneously against specific experiment condition with other widely used statistical methods.

### Data

A meta-dataset is set up by calculating the log-FC matrix from *P.aeruginosa *DNA microarray measurements diverse experimental conditions. DNA microarray datasets are retrieved from two public databases: the Gene Expression Omnibus (GEO) [23] and the ArrayExpress [24]. The measurement platform is the Affymetrix *GeneChip*^® ^Pseudomonas aeruginosa Genome Array (registered as GPL84 in GEO and A-AFFY-30 in ArrayExpress), which consists of 5883 probes (5549 protein coding genes of the PAO1 strain, 18 tRNA and rRNA of the PAO1, 117 genes from other strains and 199 intergenic sequences). We extract 5549 coding genes from 289 datasets (282 from GEO and 7 from Array- Express), which do not contain Null values (NA or missing values) or 0. RMA normalization [25] is applied to the microarray datasets in each study. Then the log-FC matrix is calculated.

### Operon genes

We use test data for evaluation of our method. Here we assess the method's performance of detection of data which should be detected and evaluate its selectivity. We focus on the operon gene, one of the biological mechanism. Operon genes which prokaryote originally have are transcripted at same time and correspond to common function [26,27]. Therefore, we think these genes must be co-expressed against specific experiment condition because of necessity of functional expression. We identify 93 operon genes in 5549 codings genes by Operon Database [28] at Kyoto University and the Pseudomonas Genome Database [29] at the University of British Columbia. When a pair of two genes is chosen from an operon, the number of all possible gene pairs is 857 for these 93 operons. Actually, Pearson's correlation coefficient of these 857 operon gene pairs is 0.734 and shows strong positive correlation, whereas that of randomly chosen gene pairs is 0.182 on the log-FC matrix. Therefore, we use operon gene as objective test data. Operon genes are not necessary to be expressed in any experimental condition. However, once some genes which belong to an operon, all the operon genes should be expressed simultaneously. Therefore, we regard operon genes which changed its expression level in specific experimental condition as correct data in the experiment condition and non-operon genes as incorrect data. Here we compare all method by evaluating how specifically detect these operon genes.

### Compared methods

We compare our two-way AIC method to other widely used DEG detection methods; *t*-rank [30] with *F*-test (experiment side in meta-dataset), RankProducts [31] (experiment side in meta-dataset), SAM (significance analysis of microarray) [32] (experiment side in meta-dataset), one side *U*- value outlier detection [19] (both gene side and experiment side in log-FC matrix), 2-*σ *(both sides simultaneously in log-FC matrix) and 3-*σ *(both sides simultaneously in log-FC matrix) (Table 1).

**Table 1:** Results of comparisons of each method's performance

Method	$\bar{se}$	$\bar{sp}$	$\bar{p}$	$\bar{nd}$	$\bar{pd}$
**1. two-way AIC **	0.58578	0.99998	2.721 × 10^-5^	5.71280	0.10295
2. *t*-rank/*F *-test	0.58477	0.99821	7.901 × 10^-3^	245	4.41521
3. RankProducts	0.58597	0.99717	1.123 × 10^-2^	312	5.62263
4. SAM	0.58690	0.99983	9.034 × 10^-4^	96	1.73004
5. *U *-value (gene side)	0.65665	0.68416	2.085 × 10^-1^	54.49481	0.98206
6. *U *-value (experiment side)	0.75034	0.99967	5.325 × 10^-4^	23.91349	0.43095
7. 2-*σ*	0.65270	0.99871	5.202 × 10^-3^	74.96886	1.35103
8. 3-*σ*	0.65488	0.99990	4.030 × 10^-4^	17.01730	0.30667

We assess the performance of all methods by calculating $\bar{se}$ (average sensitivity of detection of gene pairs in all operons), $\bar{sp}$ (average specificity of detection of gene pairs in all operons), $\bar{p}$ (average *p*-values of detection of gene pairs in all operons), $\bar{nd}$ (number of DEGs), and $\bar{pd}$ (the percentage of DEGs). Threshold of *t*-rank/*F*-test, RankProducts and SAM is set to 245, 312 and 96 to match the sensitivity with that of two-way AIC.

The judgment criterion of the *t*-rank with *F*-test, the RankProducts method and SAM is set to the rank which makes the sensitivity of these methods closest to that of the two-way AIC. In the *F*-test, we evaluate the equality of variance (*p *= 0.05), and in the case of equal variances, we calculate Student's *t*-statistic, otherwise Welch's *t*-statistic with the threshold value (upper 245 genes). The RankProducts method is a non-parametric FC based DEG detection method. We used it with the threshold value (upper 312 genes). SAM is a non-parametric *t*-statistic based DEG detection method. We used it with the threshold value (upper 96 genes).

In the 2- and 3-*σ *methods, log-FC values of genes that are larger than the threshold in both sides are detected as DEGs. The threshold is the standard deviation multiplied by 2 (2*σ *method) and 3 (3*σ *method). *σ *is calculated for each direction.

### Analyses of detected genes

The expected DEGs of each dataset in the meta- dataset mutually differ because their experimental conditions differ. Therefore we report the detection performances of the two-way AIC and other methods to show how precisely operon genes are detected simultaneously. For all pairs of detected genes (denoted by gene *a *and *b*) as DEGs by each detection method, then the pair is a "detected operon gene pairs" when there is *j *in the judgment matrix so that *x_a,j _*= *x_b,j _*≠ 0. Performance, sensitivity, specificity, *p*-value, the number and the percentage of DEGs are calculated as follows:

(4) $$\bar{se}=\frac{1}{NM}\overset{N}{\underset{k=1}{\sum }}\overset{M}{\underset{j=0}{\sum }}\frac{O_{k,j}}{T_{k}}$$

(5) $$\bar{sp}=\frac{1}{FNM}\overset{N}{\underset{k=1}{\sum }}\overset{M}{\underset{j=0}{\sum }}A_{k,j}$$

(6) $$\bar{p}=\frac{1}{NM}\overset{N}{\underset{k=1}{\sum }}\overset{M}{\underset{j=0}{\sum }}P_{k,j}$$

(7) $$\bar{nd}=\frac{1}{E}\overset{E}{\underset{j=1}{\sum }}n_{j}$$

(8) $$\bar{pd}=\frac{100}{GE}\overset{E}{\underset{j=1}{\sum }}n_{j},$$

where *N *is the number of operons in which the belonging genes were detected as DEGs at least once (0 ≤ *N *≤ 93), *M *is the number of experiments in which belonging genes were detected as DEGs at least once (0 ≤ *M *≤ 289), *O_k,j _*is the number of detected operon gene pairs, *T_k _*is the number of all possible operon gene pairs in *k*-th operon, *A_k,j _*is the number of never-detected non-operon gene pairs, *P_k,j _*is the *p*-value in the *k*-th operon, *j*-th experiment calculated using Fisher's exact test, *F *is the number of all possible combination of non-operon gene pairs (_5549_*C*_2 _- 857 = 15392069), *G *is the total number of genes (5549), *E *is the total number of all experimental conditions (289), and *n_j _*is the number of DEGs in the *j*-th experiment.

### Scalability

**Figure 5:**
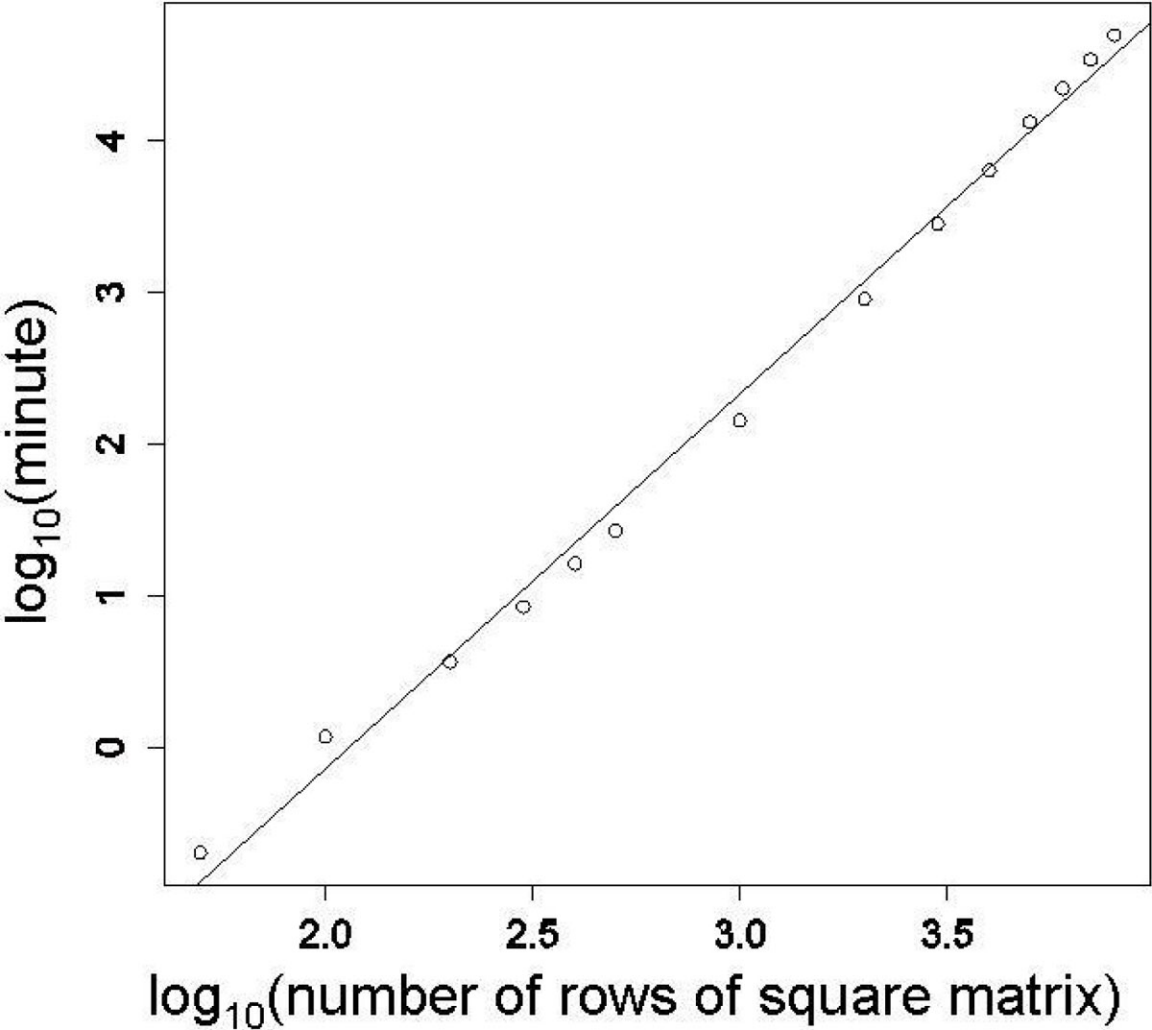
**Scalability of two-way AIC**. Scalability is assessed using some square matrices. Each element of a square matrix is set to a pseudorandom number of a normal distribution. The x-axis is the number of row (or column) of the matrix and the y-axis is the computation time in minutes necessary to finish the calculation. Both axes are transformed as logarithmic values (base 10).

Scalability of two-way AIC is assessed by some square matrices of random numbers (Figure 5). The x-axis shows the number of rows (or columns) of the square matrix. The y-axis is computation time in minutes necessary to finish the calculation. The linear regression model by the least squares method is *y *= 8.30*×*10*^-^*^6 ^· *x*^2.47^, where the coefficient of determination is 0.9946. Therefore, the calculation cost of the two-way AIC is estimated to be polynomial: O(*x*^2.47^). Computational time is measured using GNU R 2.15.0 on Mac OS × 10.6.8, 2.4 GHz Intel Core 2 Duo, and 8 GB 1067 MHz DDR3 RAM.

## Discussion

Results show that the two-way AIC is superior to all other method in *p*-value and specificity. It means that false positives of the two-way AIC is the lowest. Among other widely used methods (*t*-rank/F-test, RankProducts and SAM), SAM shows the highest specificity. However, specificity of our method is much higher than that of SAM. It suggest the effectiveness of two-way approach. Compared with other two-way method (2-*σ*, 3-*σ*), specificity of two- way AIC is also highest. It means specificity of *U*-value is superior to that of standard deviation in this case. Therefore, the two-way AIC method can detect operon genes with less noises even with all genes in an operon do not alway express proportionally [33].

Detection sensitivity is generally lower compared for specificity of all methods we tested. Compared to *U*-value method (gene side and experiment side), sensitivity of two-way AIC is not high. In general, one-way methods (*U*-value methods in Table 1) detects more operon genes than two-way methods because these methods are considered as one-pass outlier filtering while two-way methods are double filtering. However result show that double filtering cause much low false positive and choose genes that should be detected.

Any statistic including the *t*-test can be applied in two-way approach to meta-datasets in general, however, how to set the detection criterion or threshold of outliers is a major concern in these approaches. Introducing a model selection criteria AIC does not needed trial and error to find optimal threshold.

**Figure 6:**
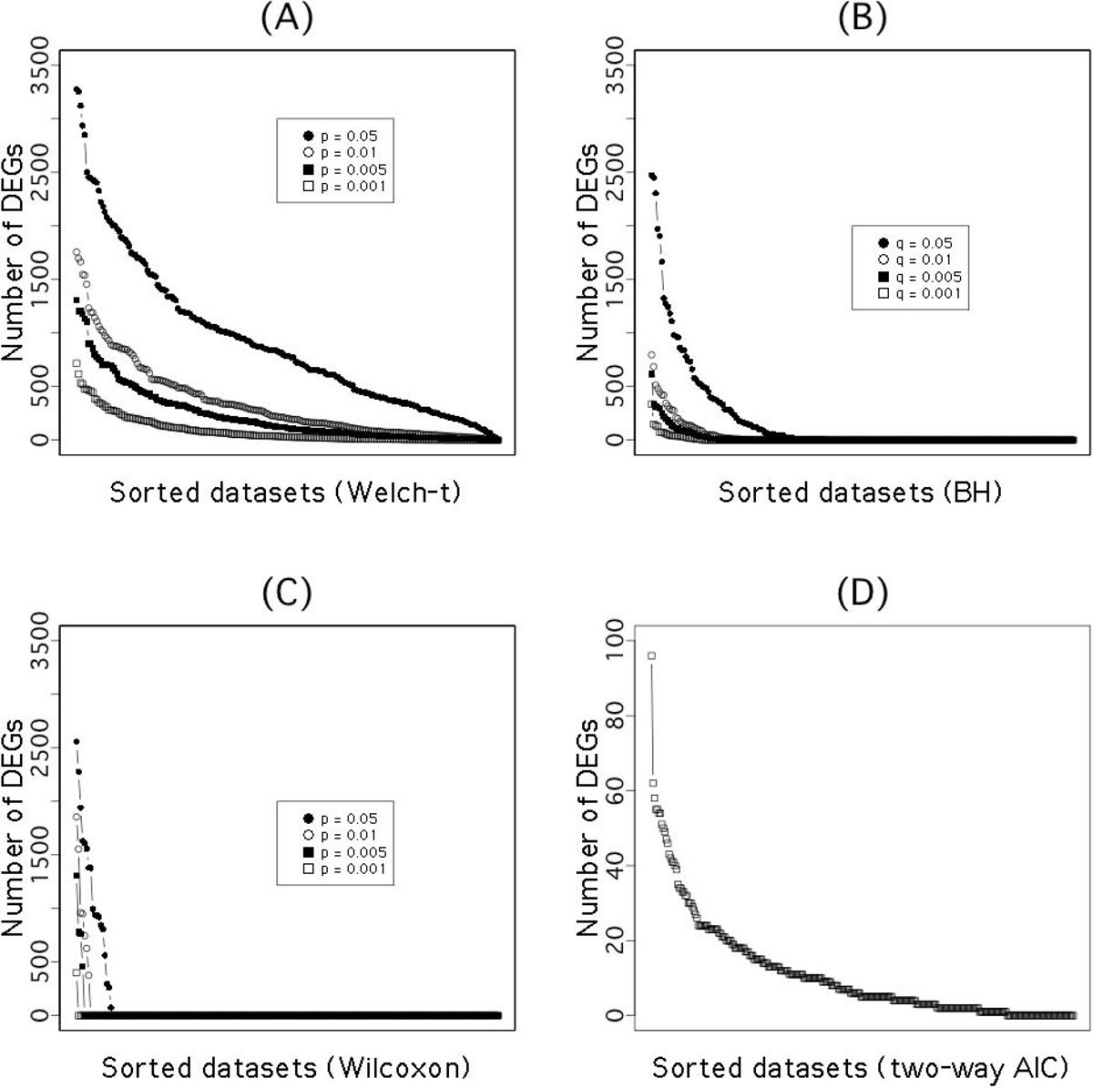
**Number of DEGs in each experiment detected by significance level**. We performed Welch *t*-test (A), BH (Benjamini-Hochberg) method with *p*-value of Welch *t*-test (B) and Wilcoxon rank sum test (C) against 209 datasets with four significance level (0.001, 0.005, 0.01, 0.05). The 209 datasets, chosen from 289 datasets in this paper consist of control and treatment groups, each of which has two or more samples so that we can calculate the variance. The x-axis is the dataset sorted by the number of DEGs. The y-axis is the number of DEGs in each dataset. Performance of our two-way AIC is shown as (D).

The stability of detection methods is shown in Figure 6. Significance level based methods (Welch's *t*-test, Benjamini-Hochberg method (BH) method [34] and Wilcoxon rank sum test often show anomalous results in which most DEGs are found in a few measurements. In the case of the Wilcoxon test, large numbers of DEGs are detected for a few experimental conditions and almost nothing is found for many conditions, and its detection results are highly variable depending on detection criteria (*p*-values of 0.05 to 0.001). It can be almost meaningless to detect DEGs from a meta-dataset that includes a wide variety of experimental conditions. Larger *p*-value or *q*-value is needed for test criteria to improve such detection of Welch's *t*-test and BH method, however, such large threshold will allow to result detecting extremely a large number of DEGs in a specific few experiments. For example, about 3000 genes are detected in Welch *t*-test with 0.05 *p*-value. Analyzing of multiple dataset uniformly by single significance level is difficult. Such situation is also found other meta-analysis study [35]. Steepness of the curve by the two-way AIC is milder than those of these methods, which means that it is less anomalous.

**Figure 7:**
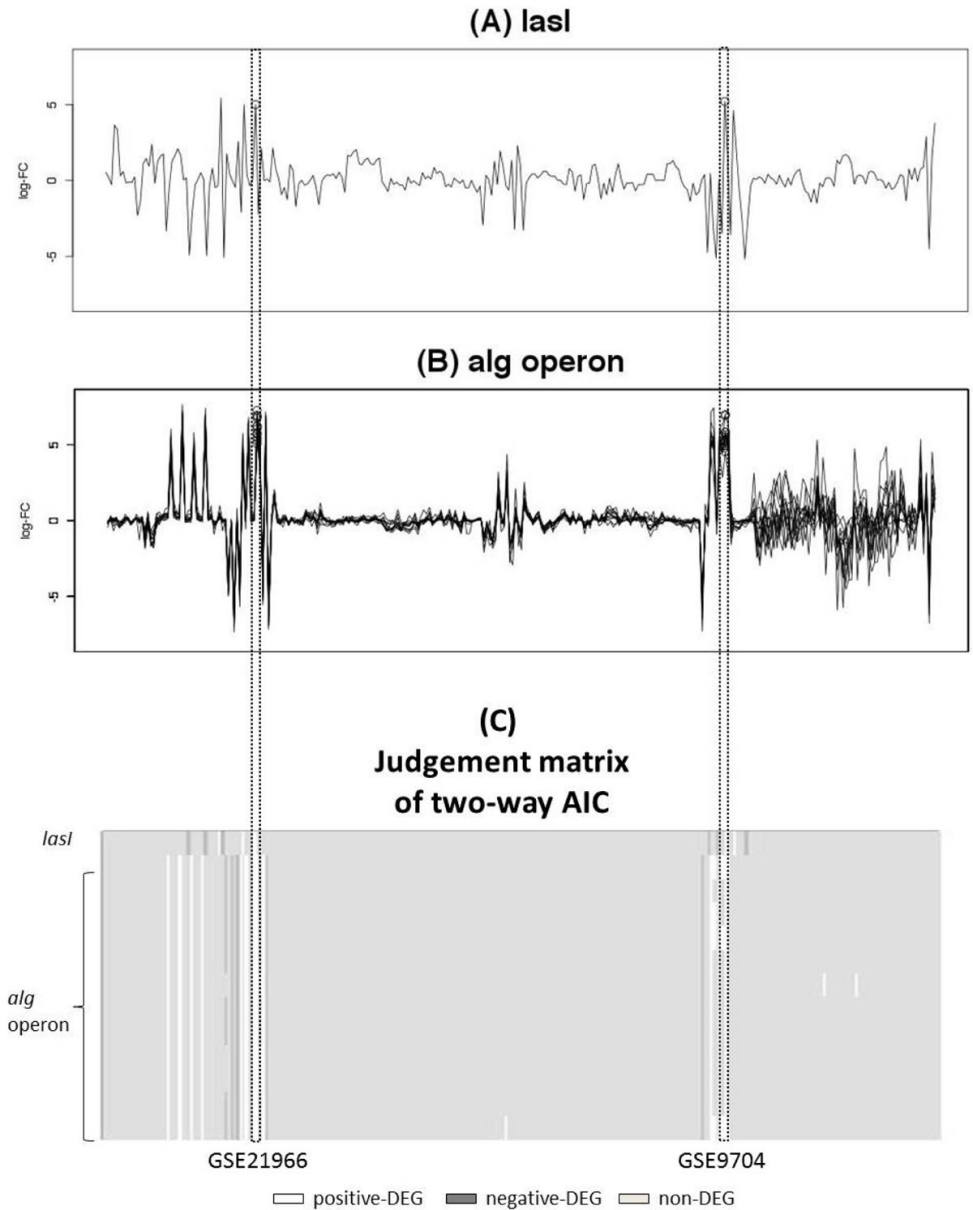
**Co-expression of ***lasI ***and ***alg ***operon**. *lasI*, one of Quorum Sensing (QS) -releated gene (A) and *alg *operon (*algD*/*alg8*/*alg44*/*algK */*algE*/*algG*/*algX*/*algL*/*algI*/*algJ*/*algF*/*algA*), biofilm-related genes (B) are detected by two-way AIC respectively. In the judgement matrix of two-way AIC, these two kind of genes are co-expressed in GSE21966 and GSE9704 (C). It is suggested that these genes are related in two experiment condition.

Finally, we show an application of our two-way AIC method to detecting mutual gene interactions. *lasI*, which is one of the QS-related gene, is suggested to regulate biofilm formation [36]. Biofilm is the mucoidy structure consisting of polysaccharide that bacteria produced. QS intervention against Biofilm formation is phenotypically observed by mutation experiment. However, its biological mechanisms such as pathway, gene regulation, molecular mechanism or other specific molecular biological evidence is still unknown [37,38]. In the judgement matrix of two-way AIC, this interaction is actually observed in two experiment condition (Figure 7) and these condition is designed by two independent researches. Both researches used *P.aeruginosa *which is isolated from Cystic Fibrosis Patients [39,40]. Actually biofilm contributes some diseases [41] and especially relationship of Cystic Fibrosis [42] is attracting attention of many researchers [43]. Interestingly, QS intervention to biofilm is not mentioned in these literatures because it is not a purpose of their experiments. However, the two-way AIC method detects a possible gene interaction which implies that *lasI *is related to biofilm formation in Cystic Fibrosis patient and perhaps *lasI *inhibition will stop biofilm formation and Cystic Fibrosis. In this way two-way AIC can help building hypothesis about mutual gene interaction across the multiple experimental condition datasets.

Supplemental material such as meta- dataset of *P.aeruginosa *and R script used in this paper are available on the web (http://www.ps.noda.tus.ac.jp/2way-aic/).

## Competing interests

The authors declare that they have no competing interests.

## Authors' contributions

KT designed the study, retrieved all data used in this work, performed the analysis, and drafted the manuscript. DT helped to design the study, to select statistical methods to be compared, to interpret the result, and to draft the manuscript. YK and SM supervised all work. All authors were involved in drafting the manuscript. They have read and approved the final manuscript.

## Declarations

The publication costs for this article were funded by the corresponding author's institution.

This article has been published as part of *BMC Genomics *Volume 14 Supplement 2, 2013: Selected articles from ISCB-Asia 2012. The full contents of the supplement are available online at http://www.biomedcentral.com/bmcgenomics/supplements/14/S2.
